# A combined strategy involving Sanger and 454 pyrosequencing increases genomic resources to aid in the management of reproduction, disease control and genetic selection in the turbot (*Scophthalmus maximus*)

**DOI:** 10.1186/1471-2164-14-180

**Published:** 2013-03-15

**Authors:** Laia Ribas, Belén G Pardo, Carlos Fernández, José Antonio Álvarez-Diós, Antonio Gómez-Tato, María Isabel Quiroga, Josep V Planas, Ariadna Sitjà-Bobadilla, Paulino Martínez, Francesc Piferrer

**Affiliations:** 1 Institut de Ciències del Mar, Consejo Superior de Investigaciones Científicas (CSIC), Barcelona, 08003, Spain; 2 Departamento de Genética. Facultad de Veterinaria, Universidad de Santiago de Compostela, Campus de Lugo, Lugo, 27002, Spain; 3 Departamento de Matemática Aplicada, Facultad de Matemáticas, Universidad de Santiago de Compostela, Santiago de Compostela, 15781, Spain; 4 Departamento de Geometría y Topología, Facultad de Matemáticas, Universidad de Santiago de Compostela, Santiago de Compostela, 15782, Spain; 5 Departamento de Ciencias Clínicas Veterinarias. Facultad de Veterinaria, Universidad de Santiago de Compostela, Campus de Lugo, Lugo, 27002, Spain; 6 Departament de Fisiologia i Immunologia, Facultat de Biologia, Universitat de Barcelona and Institut de Biomedicina de la Universitat de Barcelona (IBUB), Barcelona, Spain; 7 Instituto de Acuicultura Torre de la Sal (IATS-CSIC), Ribera de Cabanes, Castellón, 12595, Spain

**Keywords:** Turbot, NGS, Database, Transcriptome, Reproduction, Immune, Genetic Markers, SNP, Microsatellite, Microarray, Natural Antisense Transcripts, MicroRNA

## Abstract

**Background:**

Genomic resources for plant and animal species that are under exploitation primarily for human consumption are increasingly important, among other things, for understanding physiological processes and for establishing adequate genetic selection programs. Current available techniques for high-throughput sequencing have been implemented in a number of species, including fish, to obtain a proper description of the transcriptome. The objective of this study was to generate a comprehensive transcriptomic database in turbot, a highly priced farmed fish species in Europe, with potential expansion to other areas of the world, for which there are unsolved production bottlenecks, to understand better reproductive- and immune-related functions. This information is essential to implement marker assisted selection programs useful for the turbot industry.

**Results:**

Expressed sequence tags were generated by Sanger sequencing of cDNA libraries from different immune-related tissues after several parasitic challenges. The resulting database (“Turbot 2 database”) was enlarged with sequences generated from a 454 sequencing run of brain-hypophysis-gonadal axis-derived RNA obtained from turbot at different development stages. The assembly of Sanger and 454 sequences generated 52,427 consensus sequences (“Turbot 3 database”), of which 23,661 were successfully annotated. A total of 1,410 sequences were confirmed to be related to reproduction and key genes involved in sex differentiation and maturation were identified for the first time in turbot (*AR*, *AMH*, *SRY*-related genes, *CYP19A*, *ZPG*s, *STAR FSHR,* etc.). Similarly, 2,241 sequences were related to the immune system and several novel key immune genes were identified (*BCL*, *TRAF*, *NCK*, *CD28* and *TOLLIP,* among others). The number of genes of many relevant reproduction- and immune-related pathways present in the database was 50–90% of the total gene count of each pathway. In addition, 1,237 microsatellites and 7,362 single nucleotide polymorphisms (SNPs) were also compiled. Further, 2,976 putative natural antisense transcripts (NATs) including microRNAs were also identified.

**Conclusions:**

The combined sequencing strategies employed here significantly increased the turbot genomic resources available, including 34,400 novel sequences. The generated database contains a larger number of genes relevant for reproduction- and immune-associated studies, with an excellent coverage of most genes present in many relevant physiological pathways. This database also allowed the identification of many microsatellites and SNP markers that will be very useful for population and genome screening and a valuable aid in marker assisted selection programs.

## Background

The turbot (*Scophthalmus maximus*) is a flatfish with increasing commercial relevance in Europe with a current annual production of ~10,000 tones [1] with an increasing consumer demand worldwide. Thus, turbot production significantly increased in Northern China during the last decade. However, fish disease outbreaks collapsed its production in 2006, with economic losses estimated to amount several hundred million Euros [2,3].

It seems clear that one of the major concerns for turbot aquaculture is disease control. Intensive culture conditions in fish farms favors the proliferation of pathogens and the consequent economic losses associated with disease outbreaks. Hence, a comprehensive knowledge of the immune system of commercially important fish species is required [4]. The immune-prophylactic control of fish diseases through vaccination, probiotics and immunostimulation has been undertaken since long ago [5-7], whereas genetic programs on disease resistance, specifically in turbot, clearly require further investigation. Obtaining resistant broodstock is an appealing solution to control diseases in front of the economic cost of vaccines, treatments and the possible generation of resistances against antibiotics.

Another major concern for the aquaculture industry is fish reproduction. Like in other vertebrates, reproduction in turbot is controlled by the brain-pituitary-gonad axis, which integrates environmental signals and controls the production and secretion of the major hormones involved in controlling the reproductive cycle, including the onset of puberty [8,9]. Furthermore, turbot exhibits one of the largest cases of sexual dimorphism for growth rate in favor of females among aquacultured species [10]. Therefore, there is an interest in the turbot aquaculture industry to produce stocks with as many females as possible in order to increase biomass. Gonad development is a complex biological process in which an undifferentiated bipotential gonad is transformed into either a testis or an ovary [11,12] according to sex determination and differentiation [11,13]. External factors such as temperature, pH or social behavior can directly influence gonadal development in some fish [14,15] and, consequently, affect sex ratio. Understanding the process of gonadal development can greatly aid in the control of sex ratios in finfish aquaculture. However, in turbot there is a lack of information of genes involved in reproduction and their interactions. The induction of gynogenesis suggested a XX/XY system of sex determination [16], but later studies involving the analysis of progenies from sex-reversed parents revealed a ZW/ZZ system [17]. Linkage maps were developed [18-21] and led to the identification of the major sex-determining region [22] and facilitated the characterization and mapping of sex-associated markers [23,24], although the sex determining gene(s) is (are) still unknown.

Despite recent increases in the number of Expressed Sequence Tags (ESTs) for flatfish [25-28], their resources are still limited when compared to those available for salmonids (25,781 vs 851,722; GenBank). Particularly in turbot, only 12,427 entries were found but this number was recently raised up to 55,404 contigs [29]. During the last few years, efforts have been done to create a comprehensive turbot database with a large number of gene sequences available based on the immune response to the most common different pathogens of industrial relevance [30-32]. These include *Aeromonas salmonicida* subspecies *salmonicida,* a bacterium capable of causing 100% mortalities in just 7 days after challenge [33], and the parasites *Philasterides dicentrarchi* and *Enteromyxum scophthalmi*, which are responsible for severe fish outbreaks [34,35]. Therefore, the first Turbot database was originally created with almost ten thousand high-quality ESTs sequences [36] (Table 1). From this database, a first custom oligo-microarray (8X15 Agilent) was successfully designed [37] and applied for evaluating expression profiles of genes involved in defense against pathogens [38,39].

**Table 1:** **Increase of the genomic resources for the turbot ( *S. maximus *) with the successive databases**

**Turbot database**	**Source tissue**	**Sequencing strategy**	**No. of sequences (reads)**	**No. unique sequences**	**Reference**
				**(contigs + singletons)**	
1	Liver, head kidney, spleen (*Aeromonas* and *Philasterides* infection)	ABI3730 cDNA library	9,873	3,482	Pardo *et al*., 2008 [36]
				(1,073 + 2,409)	
2	Muscle	ABI3730 Microsatellite- enriched DNA library	1,371	-	Pardo *et al*. 2006 [51]
	Liver, kidney and gills (nodavirus infection and stimulation	ABI3730 cDNA library	3,339	-	Park *et al*., 2009 [52]

	Liver, head kidney, spleen, pyloric caeca and thymus (*Enteromyxum* infection)	ABI3730 cDNA library	3,043	6,170	Ribas *et al*., present work
				(1,827 + 4,343)	
3	Brain-hypophysis, gonad	454 Roche Titanium	1,191,866	52,427 + 176,451	Ribas *et al*., present work

Next Generation Sequencing (NGS) strategies have positively affected genetics research over the last few years and their advantages have been applied to many research fields. 454 FLX Titanium is a massive pyrosequencing strategy which generates medium-size single reads uncovering large amounts of DNA sequences providing much deeper sequencing coverage than it is possible with conventional Sanger sequencing [40]. Sequencing small subsets of the genome such as the transcriptome is an attractive alternative for gene discovery in species whose genome is still not available, and fish are not an exception. For example, in guppy (*Poecilia reticulata*) the sequencing of a total of 336 megabases (Mb) produced the first reference transcriptome for this fish species [41]. In the self-fertilizing hermaphroditic mangrove Rivulus, *Kryptolebias marmoratus*, the identification of more than 150,000 sequences provided the first insights on the mechanisms underlying the response to environmental stresses and chemical toxicities [42]; and in the gilthead sea bream (*Sparus aurata*), the fast skeletal muscle transcriptome was described [43]. In particular, the reproductive system of the lake sturgeon (*Acipenser fulvescens*) has also been studied by resorting to modern pyrosequencing and it has been useful for the discovery and evaluation of candidate sex-determining genes and xenobiotic-responsive genes in the gonads [44,45].

Another approach to improve the aquaculture production is based on the application of molecular markers such as microsatellites or simple sequence repeats (SSRs) and SNPs. These markers are the basis for genetic mapping and comparative genomic analysis, which are in turn used for detection of quantitative trait loci (QTL) and for marker assisted selection (MAS) programs [46,47]. Several types of genetic markers have been developed and investigated in turbot [48] and many of them have already been mapped [18-21]. Recently, a genome scan for sex-determination [22] and resistance/survival to *A. salmonicida*[49] and *P. dicentrarchi*[50] using the genetic map identified several consistent QTLs and associated markers in turbot, which suggests the existence of genetic factors underlying these characters and supports their application in genetic breeding strategies. The advents of new high-throughput sequencing technologies, which produce extensive sequence data, are providing new opportunities to increase the amount of molecular markers, as demonstrated in the sturgeon, where hundreds of SNPs were discovered [44].

Overall, the improvement of the turbot aquaculture industry by selecting, on one hand, the most resistant broodstock and, on the other hand, female-biased batches is a priority challenge. The purpose of this study was to increase turbot database information for genes related to the immune and reproductive systems by creating a powerful tool for genomic research in this species. The turbot database was updated with genes obtained both by Sanger sequencing from immune-related tissues after challenges with the myxozoan parasite *E. scophthalmi* and by a 454 FLX Titanium run from gonad and brain-hypophysis at different stages of development. Description and comparison of the two sequencing strategies, annotation procedures, and construction of a larger database, the support for microsatellites and SNP discovery, and for designing a pilot-microarray platform, are presented.

## Results and discussion

### The increase of known immune-related genes in turbot by Sanger sequencing

The progression in the construction of the turbot database is summarized in Table 1. First, the Turbot 1 database was created from almost ten thousand high-quality EST sequences from three cDNA libraries of three immune relevant organs (liver, head kidney and spleen) generated from turbot infected with *A. salmonicida* subspecies *salmonicida* and *P. dicentrarchi*, as well as from non-infected fish [36]. The Turbot 2 database included several resource sequences: i) 1,371 sequences from seven microsatellite-enriched DNA libraries from muscle tissues [51]; ii) 3,339 ESTs available in public databases [52], which were loaded on the turbot database and clustered with the set of the existing EST; and iii) Sanger sequencing data from two new cDNA libraries generated from several immune tissues (liver, head kidney, spleen, pyloric caeca and thymus) after challenging with the myxosporean parasite *E. scophthalmi* produced a total of 3,043 sequences (see Methods). Together, Sanger-based sequencing generated 17,626 sequences with an average length of 501 base pair (bp), constituting the Turbot 2 database. The assembly of all these available data consisted of 6,170 putative transcripts of which 1,827 were contigs and 4,343 singletons. A high level of redundancy was found (75.6%), which is usually observed when non-normalized cDNA libraries are used [53], but it constitutes an appropriate approach to obtain a first picture of the immune response [36].

A total of 6,053 out of the 6,170 unique sequences (98.1%) in Turbot 2 database displayed significant matches with sequences available in public databases with E-values equal or less than 1,00E-5 (6,049 with BLASTN and 2,116 with BLASTX). Gene Ontology (GO) annotation classified sequences as follows: 586 in Biological Process (BP), 472 in Cellular Component (CC) and 692 in Molecular Functions (MF).

### 454 pyrosequencing of the turbot brain-hypophysis-gonad axis transcriptome

**Figure 1:**
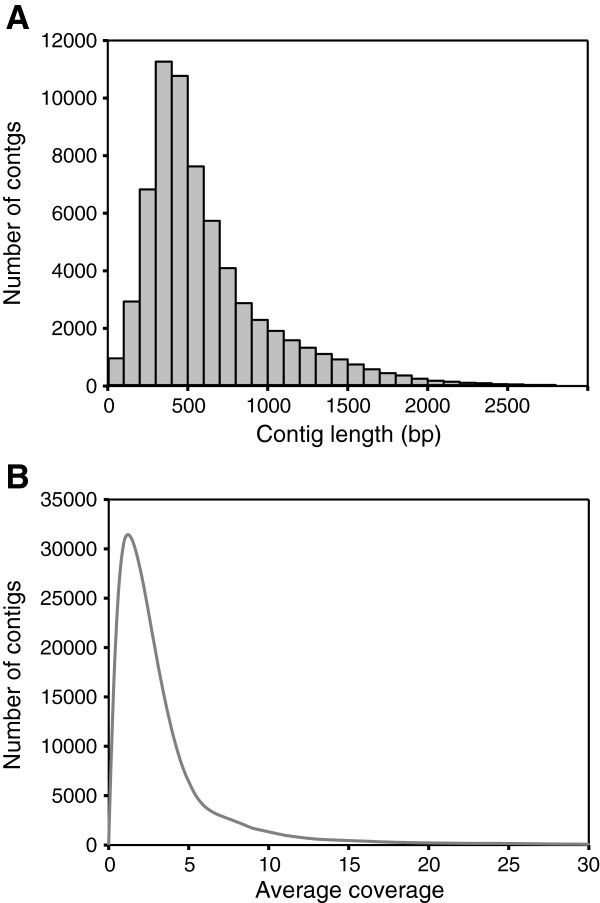
**Length range distribution of pyrosequencing contig reads of the turbot brain-hypophysis-gonad axis at different stages of gonad development.** (**A**) Range distribution of contigs obtained in the 454 FLX Titanium run. (**B**) Average coverage distribution per nucleotide of the contigs obtained in the 454 FLX Titanium run.

The Turbot 2 database described above contained 17,626 sequences, many of them related to the immune response. However, further exploration of this database revealed that sequences related to reproduction, the other major issue for turbot farming, were underrepresented. In order to obtain more sequences of genes related to sex phenotype and reproduction control, and for isolation of EST-associated genetic markers, a 454-pyrosequencing run was performed from the brain-hypophysis-gonadal axis by using tissues of 30 turbot individuals at different stages of sexual development. Table 2 summarizes the statistics of the turbot pyrosequencing normalized library. Raw data generated 2,762,845 sequences. These sequences were filtered using Roche’s software with default settings. After filtration, 1,191,866 sequence reads (341.2 megabases, Mb) were obtained with an average length of 286 bp. Sequences were assembled into 65,472 contigs (and 172,108 singletons) with a mean length of 625.9 bp (median = 499 bp; mode = 365 bp). About half of these contigs (32,612) were longer than 500 bp and their distribution by range was the highest for the 200–499 bp length, followed by the 1–199 bp length and finally by the 500–999 bp length (Figure 1A). The average depth coverage per contig was of 4.6 sequences (SD: 11.7; Figure 1B). Reads obtained in this high throughput sequence analysis have been submitted to the NCBI Sequence Read Archive under accession number SRA056483.

**Table 2:** **Summary statistics of ( *S *. *maximus*) 454-pyrosequencing**

Total reads (raw wells)	2,762,845
High quality reads (filtered)	1,191,866
Total megabases (Mb)	341.2
Average read length (bp)	286
N50 read length (bp)	383
Number of contigs	65,472
Number of contigs > 500 bp	32,612
Number of singletons	172,108
Total consensus length (Mb)	41
Average contig coverage	4.6
Maximum coverage	578.7
Mean contig length (bp)	625.9
Median contig length (bp)	499
Mode contig length (bp)	365
N25 contig length (bp)	1,217
N50 contig length (bp)	748
N75 contig length (bp)	482

Table 3 shows the top 20 longest contigs obtained from the 454 run with their annotation. They ranged from 3,550 bp to 5,012 bp and their average coverage depth per nucleotide ranged between 4.3 and 33.2. Cytochrome c oxidase subunit 3 was the longest contig. Table 4 shows the top 20 contigs with the deepest coverage (253.2–578.7). Although a normalized library was used, most contigs with the deepest coverage corresponded to protein ribosomal genes. However, genes involved in the reproductive system such as the histone deacetylase complex or the epididymal secretory protein, which is highly expressed on the surface of ejaculated spermatozoa [54], were also present.

**Table 3:** **List of the top 20 longest contigs originated from the 454 run of brain-hypophysis-gonad axis tissues of turbot ( *S *. *maximus*)**

**Contig number**	**Length**	**No. reads**	**Average coverage per nucleotide**	**% GC**	**Annotation**	**E-value**	**Accession number**	**Database**
358	5,012	584	33.2	43.4	Cytochrome c oxidase subunit 3	1.00E-127	UniRef90_C7S7B2	Uniref90
4,333	4,486	133	8.3	45.7	Adrenodoxin-like protein mitochondrial	2.00E-60	UniRef90_Q08C57	Uniref90
2,251	4,042	146	10.5	46.1	SSRU rRNA *Cavia porcellus*	3.00E-13	AAKN02033512	SSU
425	4,010	293	23.3	52.1	Aspartate/tyrosine/aromatic aminotransferase	1.00E-101	YDR111c	COG
1,894	3,992	212	13.6	44.3	unknown			
2,165	3,964	158	11.0	50.4	Ubiquitin carboxyl-terminal hydrolase	0	UniRef90_B7Z855	Uniref90
762	3,896	464	32.4	48.6	Proliferation-associated 2G4b	1.00E-165	dre:323462	KEGG
1,956	3,797	182	13.8	47.5	Novel protein (Zgc:55794)	0	UniRef90_Q1LWK5	Uniref90
9,828	3,757	54	4.3	44.1	FAM3C	9.00E-57	UniRef90_B5X712	Uniref90
12,120	3,753	63	4.3	47.0	Zgc:165446 protein	1.00E-65	UniRef90_A6H8R7	Uniref90
3,201	3,707	128	10.3	46.6	Novel protein similar to WDR44	0	dre:569045	KEGG
2,212	3,700	116	9.1	38.6	Osteocalcin	2.00E-12	UniRef90_D2XEB2	Uniref90
1,541	3,673	181	14.7	48.3	Cell division cycle	1.00E-105	UniRef90_Q6PFU4	Uniref90
2,167	3,623	173	12.3	45.1	M-phase phosphoprotein 10	1.00E-148	dre:323426	KEGG
280	3,613	287	27.3	44.9	NADH dehydrogenase subunit 5	0	UniRef90_C7S7B6	Uniref90
4,193	3,611	85	6.2	41.3	Suppressor of tumorigenicity 7 protein	1.00E-34	UniRef90_Q1RLU8	Uniref90
644	3,571	531	42.1	46.3	RAD1 homolog	1.00E-145	UniRef90_Q6P2T4	Uniref90
1,248	3,569	127	10.8	43.8	60S ribosomal protein	2.00E-45	UniRef90_P61513	Uniref90
2,845	3,555	106	8.1	43.4	NF-kappaB repressing factor	1.00E-79	gga:422370	KEGG
5,634	3,550	84	5.8	47.1	WD repeat domain phosphoinositide	0	UniRef90_Q5MNZ6	Uniref90

**Table 4:** **List of the top 20 deepest contigs originated from the 454 run of brain-hypophysis-gonad axis tissues of turbot ( *S *. *maximus*)**

**Contig number**	**Length**	**No. reads**	**Average coverage per nucleotide**	**% GC**	**Annotation**	**E-value**	**Accession Number**	**Database**
1	744	1,997	578.7	53.7	40S ribosomal protein S9	8.00E-93	UniRef90_P46781	Uniref90
19	676	1,728	541.1	45.6	Parvalbumin	4.00E-34	UniRef90_B5WX08	Uniref90
1,926	101	466	398.6	43.6	Unknown			
13	663	935	382.1	51.0	Nucleolar protein family A3	4.00E-25	UniRef90_A4IHX9	Uniref90
88	787	1,545	366.8	53.2	Similar to ribosomal protein S7	5.00E-98	UniRef90_UPI0000E801DC	Uniref90
44	701	930	336.4	56.0	Unknown			
36	709	725	291.6	51.8	NADH dehydrogenase 1 beta	8.00E-75	UniRef90_C1BWY9	Uniref90
2	1,222	1,216	289.6	46.7	Chromobox protein homolog 3	3.00E-80	UniRef90_C3KJI6	Uniref90
6	1,103	1,335	285.0	48.4	Ribosomal protein L7	1.00E-108	dre:336710	KEGG
47	675	651	277.6	51.6	Fatty acid binding protein 11a	2.00E-49	dre:447944	KEGG
3	1,275	1,250	271.4	48.0	General transcription factor IIIA	6.00E-89	dre:445389	KEGG
92	603	687	271.2	51.9	Ribosomal protein S11	9.00E-73	UniRef90_B5FX82	Uniref90
9	759	929	270.6	50.8	Histone deacetylase complex	6.00E-74	UniRef90_C3KI01	Uniref90
179	649	738	266.8	50.0	Unknown			
68	782	1,065	264.7	51.9	Ribosomal protein	1.00E-99	UniRef90_A9Z0M8	Uniref90
82	663	679	264.5	55.7	40S ribosomal protein S27a	4.00E-65	UniRef90_P68200	Uniref90
59	749	842	260.6	53.4	Ribosomal protein L12	6.00E-82	UniRef90_Q5BKW5	Uniref90
31	869	973	258.7	54.0	60S ribosomal protein L13	1.00E-101	UniRef90_B5DGD9	Uniref90
15	997	950	255.3	51.6	Ferritin	1.00E-90	UniRef90_Q4SBB8	Uniref90
4	1,346	1,340	253.2	42.6	Epididymal secretory protein E1	3.00E-70	UniRef90_C3KIM5	Uniref90

About half of the contigs obtained in the 454 run were successfully annotated and classified into Gene Ontology categories. More precisely, contigs exclusively obtained by the 454 run were functionally classified in the BP (8,390), CC (7,081) and MF (10,026) categories.

### Creation of the turbot 3 database

The sequencing strategies used, i.e. traditional Sanger and high throughput 454, yielded a high amount of transcriptomic sequences both from immune and reproductive systems in turbot. With all the information generated, a new Turbot 3 database was created and stored in a web-based portal for exploitation, first by the consortium participating in this project and then publically once the project is finished by the end of 2013. Cap3 software was used to assemble the sequences coming from all Sanger-based libraries (17,626) and the contigs from 454-pyrosequencing (65,472), yielding 52,427 unique sequences (47,134 only-454, 2,904 mixed Sanger-454 and 2,389 only-Sanger), thus reducing redundancy among sequences (Table 1). The number of sequences generated in one single pyrosequencing run (65,472) was almost four times higher than the six libraries sequenced by Sanger together (17,626). When comparing to public turbot resources, our strategy allowed increasing by 34,400 the number of novel sequences identified for the first time in turbot.

### Annotation of the turbot 3 database

**Figure 2:**
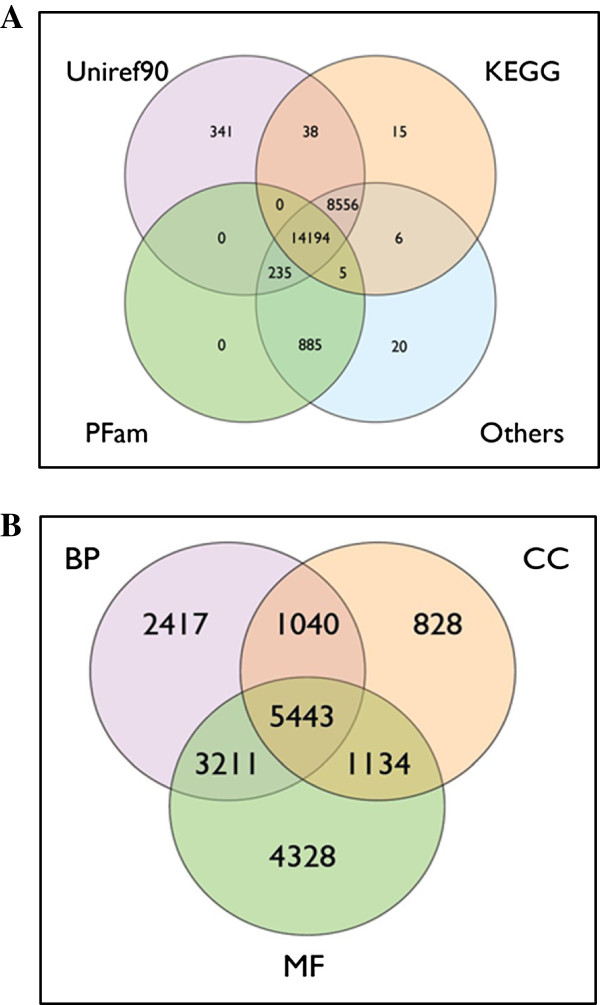
**Venn diagrams showing annotation (A) and functional classification by Gene Ontology terms (B) in the Turbot 3 database.** BP: Biological Process, CC: Cellular Component, MF: Molecular Function.

Nearly half of the sequences 23,661/52,427 (20,630 only-454, 1,969 mixed Sanger-454 and 1,062 only-Sanger) were automatically annotated by AutoFact (http://www.bch.umontreal.ca/Software/AutoFACT.htm) and produced a significant BLAST hit against at least one of the public databases. A Venn diagram showing the number of sequences that matched with some of the commonly used databases is shown in Figure 2A. A total of 14,194 sequences shared significant BLAST hit against all databases including UniRef90, KEGG, PFam and others (SSU, LSU, COG, Smart), while 8,556 contigs shared BLAST-hits against UniRef90, KEGG and other databases and 885 with PFam and other databases. About 2/3 of the contigs (18,031) were successfully classified into Gene Ontology categories: 12,111 according to BP, 8,445 to CC and 14,116 to MF categories (Figure 2B). The number of sequences exclusively assigned to each functional category was 2,417 for BP, 828 for CC and 4,328 for MF.

**Figure 3:**
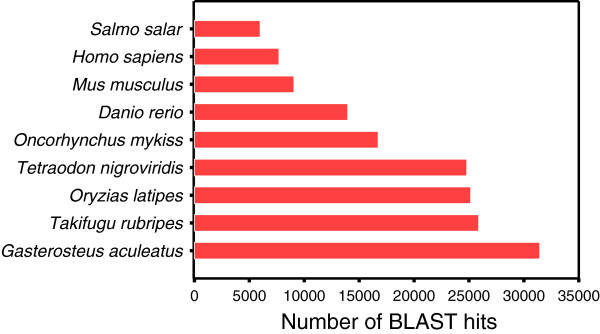
BLASTx top-hit species distribution of gene annotations in the Turbot 3 database.

Most significant BLAST-hits were obtained against a small number of species represented in public databases including model fish species (*Danio rerio*, *Tetraodon nigroviridis*, *Oryzias latipes*, *Takifugu rubripes* and *Gasterosteus aculeatus*), cultured fish species (*Oncorhynchus mykiss* and *Salmo salar*) and two mammalian species (*Mus musculus* and *Homo sapiens*) (Figure 3). *G. aculeatus* was the highest represented species followed by a group including *T. rubripes*, *O. latipes and T. nigroviridis*, all these species and turbot belonging to the Acanthopterygii superorder [55].

**Figure 4:**
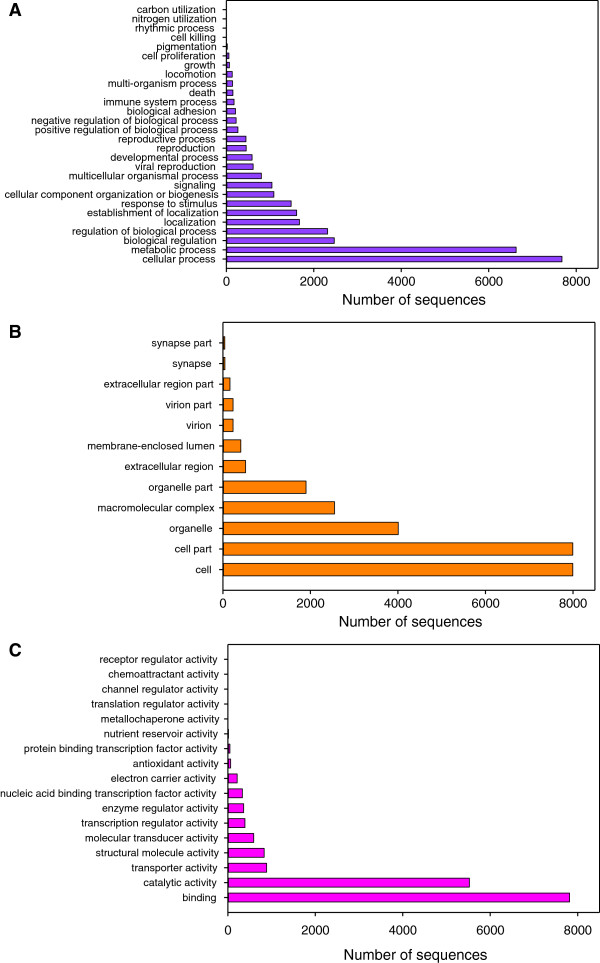
**Second level Gene Ontology assignment of sequences in Turbot 3 database. A**) Biological process; **B**) Cellular component and **C**) Molecular function.

Figure 4 summarizes the number of sequences representing the different 2nd level GO terms in the Turbot 3 database. Cellular process (7,676 sequences) and Metabolic process (6,627 sequences) were the most represented categories within BP terms (Figure 4A), but categories related to immune function had also a high representation: Response to stimulus (1,482 sequences), Viral reproduction (612 sequences), Immune system process (176 sequences) and Death (149 sequences). The reproductive system was also represented by the Reproduction (453 sequences) and Reproductive process (448 sequences) categories, and to a lower extent by Growth and Cell proliferation. Cell and Cell parts categories (7,995 sequences each) followed by Organelle (4,006 sequences) were the highest represented within CC terms (Figure 4B). Finally, within MF terms Binding (7,813 sequences) and Catalytic activity (5,523 sequences) were the most represented categories followed by Transporter activity (887) and Structural molecule activity (830) (Figure 4C).

### Identification of genes related to the immune response

The knowledge of the immune system of fish has greatly increased recently. However, there are still many fish diseases which produce important losses to industry because still there is no an effective strategy for their control, including vaccines. The immune system of fish is composed of non-specific and specific immune defenses, being the first more important than in higher vertebrates [56,57]. Examples of innate immunity include anatomic barriers, mechanical removal of pathogens, bacterial antagonism, pattern-recognition receptors, antigen-nonspecific defense compounds, the complement pathway, phagocytosis, and inflammation [58]. In the present study, the main organs of the immune system of fish such as head kidney (equivalent to the bone marrow of mammals), spleen and thymus (often neglected in fish immune transcriptomics) were included. In addition, other organs such as the liver, a multifunctional organ with innate immune functions in mammals [59] and poorly studied in fish, and the pyloric caeca, the target organ of the myxozoan parasite, which also plays a role in immunity [60], were included as well.

Next-generation pyrosequencing has become an important tool for transcriptomic studies, enabling the identification of new immune molecules that are expressed upon activation of the immune response. A remarkable recent example is the study of the liver transcriptome of orange-spotted grouper (*Epinephelus coioides*) after virus infection [61]. It seems very likely that developments related to fish immunology will have a significant impact for obtaining a new generation of vaccines against diseases. A disadvantage of turbot is that neither the genome nor the complete transcriptome are available yet and, therefore, important information about immunity and stress related genes and their expression is lacking. Many genes were identified previously in turbot using classical Sanger sequencing in response to *A. salmonicida* and *P. dicentrarchi*[36], *Vibrio harveyi*[62] and nodavirus [52]. However, the number of genes related to the immune system in this species remained low. Recently, Pereiro *et al*. [29] used 454-pyrosequencing after different immune stimulations to provide a rich source of data to improve the knowledge of *S. maximus* immune transcriptome. Their results revealed a large number of contigs and singletons with potential immune function in turbot and identified many of the proteins involved in the main immune-pathways in humans, showing the potential of pyrosequencing. Although our 454 run was not specifically from immune-related tissues, after combining the Sanger and pyrosequencing data, a significant number of genes associated to essential functions directly or indirectly related to innate and acquired immunity were detected in the Turbot 3 database (2,241 of the 23,661 annotated sequences; see Additional file 1). Most of the immune-related sequences (1,873) were derived exclusively from the 454 run and only 149 and 219 sequences from Sanger or mixed Sanger-454, respectively. We found several novel genes, including components or family members related to acute phase response and inflammation, stress and/or defense response and in the coagulation cascade. Many of the genes shown in the immune pathways presented by Pereiro *et al.*[29] (complement, Toll-like receptor signaling, B cell receptor signaling, T cell receptor signaling and apoptosis cascade) could be identified, but also some other important immune genes were identified here for the first time in turbot, a selection of which is shown in Table 5. Relevant examples include *DFF40* subunit, a substrate for caspase-3, which triggers DNA fragmentation during apoptosis; *BCL-XL*, an anti-apoptotic protein; *TRAF2*, which regulates activation of *NF-kappa-B* and *JNK*, playing a central role in the regulation of cell survival and apoptosis; *TRAF6*, which mediates signaling from members of the *TNF* receptor superfamily as well as the *TOLL/IL-1* family; *IRAK1*, which plays a critical role in initiating innate immune response against foreign pathogens; *JNK*, involved in cell differentiation and proliferation, neurodegeneration, inflammatory conditions and *AP-1-*mediated cytokine production; *TOLLIP*, which is involved in the turnover of *IL1R*-associated kinase, interleukin-1 receptor (*IL1R*) trafficking and regulation of the imflammatory signaling; *FYN*, which participates in the downstream signaling pathways that lead to T-cell differentiation and proliferation following T-cell receptor (*TCR*) stimulation; *NCK*, which plays a pivotal role in the T cell receptor (*TCR*)-induced reorganization of the actin cytoskeleton and the formation of the immunological synapse; *DLG1*, which is involved in lymphocyte activation; *COT*, which promotes the production of *TNF*-alpha and *IL-2* during T lymphocyte activation; *CD28*, involved in T-cell activation; *GADS*, involved in B and T cell activation; and *GRB2*, which provides a critical link between cell surface growth factor receptors and the Ras signaling pathway. The obtained data complements and expands the known spectrum of immunity related genes and provides a valuable platform for more detailed analyses of the immune response in fish in general a turbot in particular.

**Table 5:** Selection of some of the novel relevant immune-related genes identified in the Turbot 3 database

**Gene name**	**Gene abbreviation**	**Contig length**	**Gene Ontology**	**E-value**	**Species**	**Annotation resource**
DNA fragmentation factor, 40kDa, beta polypeptide	*DFF40*	607	GO:0006917 GO:0006309 GO:0030263	2,00E-73	*Danio rerio*	Uniref90
BCL2-like 1	*BCL-XL*	698	GO:0045087 GO:0045768	6,00E-19	*Osmerus mordax*	nr
Tumor necrosis factor receptor-associated factor 2	*TRAF2*	528	GO:0045087 GO:0008624 GO:0050870 GO:0042981	2,00E-53	*Oplegnathus fasciatus*	Uniref90
Interleukin 1 receptor activated kinase 1	*IRAK1*	842	GO:0045087 GO:0008063 GO:0006916	6,00E-28	*Siniperca chuatsi*	Uniref90
JNK1/MAPK8-associated membrane protein	*JNK1/MAPK8*	223	GO:0006986 GO:0030433	8,00E-23		Uniref90
Toll-interacting protein	*TOLLIP*	401	GO:0045087 GO:0006954 GO:0045321	2,00E-55	*Salmo salar*	nr
TNF receptor-associated factor 6	*TRAF6*	187	GO:0045087 GO:0006915 GO:0008063 GO:0050852	5,00E-15	*Gasterosteus aculeatus*	nr
FYN oncogene related to SRC, FGR, YES	*FYN*	467	GO:0006468 GO:0016310	1,00E-74	*Danio rerio*	nr
Cytoplasmic protein 1	*NCK1*	382	GO:0042110 GO:0050852 GO:0042102	4,00E-38	*Danio rerio*	nr
Cytoplasmic protein NCK2	*NCK2*	779	GO:0042102 GO:0042110	3,00E-12	Amniota	Uniref90
Disks large homolog 1	*DLG1*	402	GO:0070830 GO:0044419	2,00E-09	*Xenopus tropicalis*	nr
Mitogen-activated protein kinase 8	*COT*	705	GO:0031295 GO:0000165 GO:0000186	1,00E-07	*Tetraodon nigroviridis*	Uniref90
T-cell-specific surface glycoprotein CD28	*CD28*	689	GO:0031295 GO:0006959 GO:0008624 GO:0042102 GO:0045768 GO:0002863 GO:0045086 GO:0045066	2,00E-18	*Salmo salar*	nr
GRB2-related adaptor protein 2	*GADS*	721	GO:0031295 GO:0050852 GO:0007265 GO:0007267	5,00E-24	*Tetraodon nigroviridis*	Uniref90
Growth factor receptor-bound protein 2	*GRB2*	370	GO:0031295 GO:0050900 GO:2000379 GO:0030168 GO:0007265	3,00E-17	*Oreochromis niloticus*	nr

Several immune related pathways were also identified in the Turbot 3 database. Chemokine signaling is an important immune pathway due to the fundamental role of chemokines in providing directional cues for the trafficking of leukocytes to sites of inflammation but also it has been implicated in dendritic cell maturation, macrophage activation, neutrophil degranulation, B cell antibody class switching, and T cell activation. The data infers that chemokines influence both the innate and acquired phase of an immune response to host insults. Thus, the protein richness of this pathway in the Turbot 3 database was described (see Additional file 2). Most members intervening in this pathway were identified showing the usefulness of the Turbot 3 database for gene discovery.

### Identification of genes related to reproduction

To date, fish gonad-related ESTs are poorly represented in public databases. A first attempt to identify genes related to gonad development in male and female turbot was carried out by cDNA-AFLP technology and several specific sequences could be identified [63]. However, the amount of information presently available is still scarce and thus a small number of sex-specific sequences have been identified. Here, the use of the 454 FLX Titanium sequencing allowed obtaining a large number of gene sequences (65,472 contigs) and their subsequent assembly and gene annotation led to the identification of a total of 1,410 annotated sequences related to reproductive function. This means that sequences corresponding to many genes of the brain-hypophysis-gonad axis, expressed first during the process of sex differentiation and then during gonadal maturation, have been identified (Additional file 3). Functional annotation terms classified all those sequences in a total of 8,425 GO terms. This is the first time that sex-related genes have been massively identified towards understanding gonad development and maturation in the turbot.

Table 6 shows 20 relevant genes with a well-known function in the reproductive system, most of them identified for the first time in turbot. The annotation, as shown by the low E-values, was reliable for all of them. Some of these are genes involved in testicular development, such as the androgen receptor-alpha (*AR*), Müllerian inhibiting substance (*AMH*), *SRY*-related genes containing a HMG box (*SOX*), Spermatogenesis associated 13 or steroid 11-β-hydroxylase. *AR* has been cloned in several cultured fish species like the rainbow trout (*Oncorhynchus mykiss*) [64] and the European sea bass (*Dicentrarchus labrax)*[65]. *AR* mediates the androgen effects by binding to specific DNA recognition sites and regulating the transcription of many different processes [66]. In fish, *AMH* expression levels are consistently higher in males than in females during sex differentiation, suggesting that this factor plays an important role in testicular development [67]. The *SOX* gene family encodes an important group of developmental regulators, involved in sex determination in fish [68,69] and other key processes such as development of the central nervous system [70]. Furthermore, the important transcription factors *SOX6* and the *SOX9* were identified.

**Table 6:** Selection of some of the novel relevant reproductive-related genes identified in the Turbot 3 database

**Gene name**	**Gene abbreviation**	**Contig length**	**Gene Ontology**	**E-value**	**Species**	**Annotation resource**
Androgen receptor alpha	ARA	1,780	GO:0005634 GO:0003707 GO:0003700 GO:0006355	1,00E-110	*Gasterosteus aculeatus*	Uniref90
Cytochrome P450 aromatase	CYP19A	2,109	GO:0009055 GO:0020037 GO:0005506 GO:0004497	0	*Paralichthys olivaceus*	Uniref90
Follicle stimulating hormone receptor	FSHR	2,890	GO:0016021 GO:0007186	0	*Dicentrarchus labrax*	Uniref90
Gonadal soma derived factor	GSF	2,035	GO:0008083	3,00E-60	*Oryzias latipes*	Uniref90
Gonadotropin alpha	GTC	1,529	GO:0005576 GO:0005179	2,00E-47	*Amphiprion melanopus*	Uniref90
Growth differentiation factor 9	GDF9	2,407	GO:0008083	1,00E-131	*Dicentrarchus labrax*	Uniref90
Meiotic nuclear division protein 1 homolog	MND1	1,386		4,00E-96	*Anoplopoma fimbria*	Uniref90
Mitotic arrest deficient 2	MAD2	396	GO:0007067	2,00E-26	*Oryzias latipes*	Uniref90
Müllerian inihibiting substance	AMH	1,388	GO:0008083 GO:0008406	2,00E-47	*Paralichthys olivaceus*	Uniref90
Pituitary tumor-transforming	PTTG1IP	1,257	GO:0008083	4,00E-51	*Salmo salar*	Uniref90
Sex hormone binding globulin	SHBG	906		1,00E-70	*Verasper moseri*	Uniref90
SRY-box containing gene 6	SOX6	804	GO:0005634 GO:0003677	1,00E-83	*Takifugu rubripes*	nr
SRY-box containing gene 9	SOX9	610	GO:0005634 GO:0003677	2,00E-59	*Takifugu rubripes*	nr
Spermatogenesis associated 13	SPATA13	1,369	GO:0005089 GO:0005622 GO:0035023	1,00E-146	*Takifugu rubripes*	nr
StAR-related lipid transfer protein 5	START-5	737	GO:0006694 GO:0015485 GO:0017127	2,00E-81	*Salmo salar*	Uniref90
StAR-related lipid transfer protein 7	START-7	603		4,00E-48	*Salmo salar*	nr
Steroid 11-beta-hydroxylase	CYP11B	584	GO:0009055 GO:0020037 GO:0005506 GO:0004497	6,00E-53	*Dicentrarchus labrax*	Uniref90
Vasa	VASA	2,027	GO:0005524 GO:0008026	1,00E-128	*Auxis thazard*	Uniref90
Zona pellucida glycoproteins	ZPC	468		2,00E-14	*Sparus aurata*	Uniref90
Zygote arrest protein 1	ZAR1	353	GO:0005737 GO:0007275	4,00E-22	*Takifugu rubripes*	Uniref90

Another group of identified genes in turbot is involved in ovarian development. This is the case of the cytochrome P450 aromatase (*CYP19A*), Zona pellucida glycoprotein (*ZPG*) or the Zygote arrest protein. *CYP19A* is a key enzyme in the hormonal steroidogenic pathway that mediates the conversion of androgens into estrogens [71], with two isoforms with specific regulation and tissue distribution [72,73] and it has been cloned in several cultured fish species like European sea bass [74]. Higher expression of *CYP19A* is found in female gonads when compared to male gonads from early development and recently it has been shown that different methylation levels of its promoter are related to temperature during the thermal sensitive period [75]. *ZPG*s are glycoproteins found in the fish chorion, which mediate species-specific sperm binding [76]. *ZPG*s are encoded by multiple gene families and here several of them have been identified (*ZPG2*, *3*, *4*). The steroidogenic acute regulatory protein (*STAR*) and similar proteins containing *STAR*-related lipid transfer (*START*) are responsible for the synthesis of sex steroids and other hormones like cortisol in response to specific stimuli [77]. Here, we were able to identify two different *START* genes, *START*5 and *START 7*.

Gonadotropins (GtHs) control the complex endocrine system that regulates gonadal growth, sexual development and reproductive function, and are secreted by the hypophysis [78]. Three forms of *GnRH* in the brain and pituitary of the turbot have been identified so far [79]. One of the main gonadotropins in vertebrates, as well in fish, is the follicle-stimulating hormone (*FSH*). Its receptor, *FSHR*, is found in male and female gonads and although cloned in other cultured fishes such as the sea bass [80], here is it identified for the first time in turbot.

Not only genes expressed in somatic cells but also genes expressed in the germ cell line were present in the Turbot 3 database. *VASA* plays an important role in germ cell determination and development and is an essential factor for primordial germ cell formation and migration to the germinal ridge [81]. In fish, *VASA* was first cloned in zebrafish [82] and later in rainbow trout [83] but also in turbot (JX235364).

Table 7 shows the pathways related to reproduction identified in the Turbot 3 database with more than 50% coverage. Here, pathways are ranked based on the number of genes included in the database with respect to the total number of genes present in each pathway: Oocyte meiosis (dre04114), Circadian rhythm (dre04710), mTOR signaling pathway (dre04150), ErB signaling pathway (dre04012), Progesterone-mediated oocyte maturation (dre04914), GnRH signaling pathway (dre04912), Insulin signaling pathway (dre04910), Androgen and estrogen metabolism (dre00150), Steroid biosynthesis (dre00100), Wnt signaling pathway (dre04310), and Notch signaling pathway (dre04330). Additional file 4 shows the Progesterone-mediated oocyte maturation pathway highlighting the presence and absence of proteins in the Turbot 3 database. The exposure to either insulin-like growth factor 1 (*IGF-1*) or the steroid hormone progesterone breaks oocyte meiotic cell division arrest and induces meiosis resumption and therefore the transformation of the oocyte into a mature, fertilizable egg [84]. Oocyte maturation is also dependent on the activation of a cascade of genes, which activate the *MAPK* (mitogen-activated protein kinase) signaling pathway [85]. The key activity driving meiotic progression is the maturation-promoting factor (*MPF *), a heterodimer of *CDC2* (cell division cycle 2 kinase) and *CYCLIN-B*[86]. In fish, the importance of some of the genes involved in the oocyte maturation pathway has been described so far [87]. Here, 79 out of 105 genes belonging to this pathway were found, showing the coverage of the generated Turbot 3 database.

**Table 7:** Representation of reproductive pathways with more than 50% of coverage in the Turbot 3 database

**Description**	**KEGG code**	**No. of genes in the pathway**	**Nº of genes in the database**	**% coverage**
Oocyte meiosis	dre04114	137	114	83.2
Circadian rhythm	dre04710	30	24	80.0
mTOR signaling pathway	dre04150	60	46	76.7
ErbB signaling pathway	dre04012	102	77	75.5
Progesterone-mediated oocyte maturation	dre04914	105	79	75.2
GnRH signaling pathway	dre04912	121	91	75.2
Insulin signaling pathway	dre04910	163	117	71.8
Wnt signaling pathway	dre04310	187	121	64.7
Steroid Biosynthesis	dre00100	19	12	63.2
Notch signaling pathway	dre04330	55	31	56.4

Overall, our results show that the approach followed was successful since most of the well-known reproduction-related genes found in other species have been also identified in turbot essentially at once.

### Genetic markers

An important emerging application of high-throughput 454 sequencing is the identification of molecular markers from genomic DNA. In fact, recent studies have identified 26 polymorphic microsatellite by pyrosequencing in an endangered fish species of China [88] and 21 microsatellites loci from the threatened freshwater Yarra pygmy perch (*Nannoperca obscura*) [89]. However, few studies have been conducted to search for cDNA-associated microsatellites, like those identified in the Atlantic herring (*Clupea harengus*) [90], despite the potential for targeting candidate genes [91].

Due to their location within genes, EST-SSR markers frequently display a high degree of transferability between related species, thus facilitating comparative genomics strategies with model species. In addition, high sequence coverage in principle allows the assessment of variability *in silico*, aiding for selection of polymorphic markers. We searched for new microsatellite markers within our sequence database to identify sequences with different repeat motifs. Our search revealed 993 sequences containing 1,237 new SSRs identified from 52,427 sequences, with 394 EST sequences containing at least two SSRs. Of these, 759 showed significant hits in BLAST with an E-value cut-off of ≤ 1,00E-5 and, thus, were annotated. The frequency of EST-SSRs observed in the turbot transcriptome was 1.9%, and the distribution density was 1.48 microsatellites per Mb. SSR motifs were identified using criteria based in a minimum number of repeats for di-, tri-, tetra- or pentanucleotide motifs (see Methods section). Similar to other vertebrate genomes, the most abundant repeat type was AC (20.93%, 259) followed by AAG (15.04%, 186), AGG (10.51%, 130), AGC (9.30%, 115), and AG (6.87%, 85). The frequency of microsatellites was inverted regarding the length of the motif, dinucleotide microsatellites being the commonest ones and pentanucleotides the less abundant (Table 8). In addition, those microsatellites with a lower number of repeats were more frequent than those with a higher number of repeats, the most common class being n = 4 (438 loci: 35.41%). Further, 12.53% of loci contained more than 10 repeat units. All the new microsatellite-containing ESTs showed sufficient flanking sequence length for primer design, and 5,609 polymorphisms of them appeared polymorphic after *in silico* analysis.

**Table 8:** Frequency distribution of the new SSRs by motif length in the Turbot 3 database

	**Repeat unit number**										
**SSR motif length**	**4**	**5**	**6**	**7**	**8**	**9**	**10**	**>10**	**Total**	**% excluding 4 and 5 repetitions**	**%**
Di	-	-	98	60	33	42	33	125	391	60,7	31.6
Tri	392	136	85	46	30	23	17	27	756	35,4	61.1
Tetra	36	17	10	5	2	2	1	2	75	3,4	6.1
Penta	10	2	1	1	0	0	0	1	15	0,5	1.2
Total	438	155	194	112	65	67	51	155	1,237	100	100
%	35.4	12.5	15.7	9.1	5.3	5.4	4.1	12.5	100		

A total of 7,362 SNPs were detected in 1,040 of the 9,495 contigs using the three filters set in the QualitySNP pipeline [92]. Only clusters with at least 4 EST sequences (2,456) were selected to minimize the detection of SNPs caused by sequencing errors. On average, one SNP per 196 bp was identified, which is a frequency in the order of that estimated in non-model species [93]. The types of detected SNPs according to different criteria are summarized in Table 9. Among them, 2,223 were transitions, 2,404 transversions and 1,578 indels. In addition, the majority of SNPs were detected in contigs involving a large number of sequences, which provides an additional support for their confidence.

**Table 9:** Summary statistics of SNPs in the Turbot 3 database

	**No. Contigs**	**No. SNPs**
Total number SNPs		7,030
Total contigs with SNPs		1,040
with 4 sequences	131	270
with 5-10 sequences	620	2,516
with 11-20 sequences	147	926
with 21-30 sequences	63	859
with 31-50 sequences	40	940
with > 50 sequences	39	1,859
Total number of transitions		2,223
C/T		1,328
A/G		895
Total number of transversions		2,404
A/T		500
A/C		746
T/G		614
C/G		544
Total number of indels		1,578
Tri-allelicpolymorphisms		1,044
Tetra-allelicpolymorphisms		113

The large amount of potential molecular markers found in this study will enable more detailed population and applied genomic studies. Since these new markers are linked to genes, they will be useful as Type I markers for population genomics screening in this species and for comparative mapping and fish evolutionary studies [94].

### Pilot microarray and identification of natural antisense transcripts

To date, several custom microarrays have been designed in several non-model fish species. Examples exist in rainbow trout [95] gilthead sea bream [96], European sea bass [97], Atlantic salmon (*Salmo salar*) [98], common carp (*Cyprinus carpio*) [99] or Senegalese sole (*Solea senegalensis*) [26], but also in the turbot [37]. In the present study, samples from the reproductive and immune tissues were used to characterize their transcriptome using different sequencing strategies and *de novo* assembly to identify a large number of genes previously unknown in turbot. The assembled data present in the Turbot 3 database was the basis to construct a pilot microarray towards a new gene-enriched updated version. One of the drawbacks of 454 sequencing technology is that it may produce false annotations of genes [100,101], and since sequencing is not oriented as in cDNA libraries used for Sanger sequencing, it is not possible to know the DNA sense strand of a gene unless it is confidently annotated. To solve these problems, and in order to identify the most reliable oligos for a definitive turbot microarray, a pilot microarray was developed. In this pilot microarray, oligos were designed both in forward and reverse sequence orientation. In addition, several filtration criteria were followed to analyze microarray data (see Methods). This strategy allows, on one hand, to identify the sense strand of the non-annotated sequences, but also to identify false annotation of genes. On the other hand, this procedure also allows studying the frequency of putative natural antisense transcripts (NATs) in turbot transcriptome [97].

The importance of NATs, which can regulate eukaryotic gene expression, has emerged in the last decade [102]. A NAT is a single-stranded RNA sequence complementary to messenger RNA and includes various classes of short RNAs including micro RNAs (miRNAs), promoter-associated transcripts and long non-protein-coding RNAs [103,104]. The amount of NATs in eukaryotic cells remains unclear. It had been reported that over 20% of human transcripts might form sense–antisense pairs [105], but large-scale cDNA sequencing suggested that antisense transcription is more common than previously thought [106]. Recently, it has been shown that up to 72% of the transcripts had antisense partners in human and mouse transcriptomes [107,108]. High-throughput sequencing strategies have revealed a plethora of non-protein-coding transcripts from both genic and intergenic regions [40]. Data on miRNAs, one of the short NAT classes, has been already published in rainbow trout [109] and halibut *Hippoglussus hippoglossus*[110]. Due to their increasing importance, the study of NATs cannot be longer ignored in transcriptome studies.

The functionality of the oligos included in the pilot-microarray was checked by hybridizing the same RNA used for the Sanger and 454 sequencing strategies. To analyze microarray data two filtration criteria were applied (see Methods). Once the first filtration process was completed 37,759 signals in forward and 33,489 in reverse oligos still remained (75%) (Table 10). Then, a second filtration with two additional filtering criteria (threshold intensity <100 and cross-hybridization between oligos) was performed to select the best performing oligo probes. As seen after this additional filtration process (Table 10), among the 94,582 probes there were 53,534 (56.6%) with no signal (24,083 forward and 29,451 reverse). After the two rounds of filtration, a total of 41,048 remaining oligos (43.4%) yielded signal in at least one tissue (brain-hypophysis-gonad or immune tissues) or in both. As a result of this filtration strategy, the remaining oligos were selected to be included in the updated turbot microarray. In the development of a custom microarray for the European sea bass, a similar strategy was followed to study NATs expression [97]. Although a lesser amount of sequences was designed for this purpose (640 sequences), identification of NATs was also achieved [97].

**Table 10:** Filtration process results for the 47,921 sequences with oligos in forward and reverse orientation

	**Forward**					
	**1**^**st **^**filtration**	**Both systems**	**Only immune**	**Only reproduction**	**Without signal**	**Total**
Reverse	Both systems	12,189	275	4,297	5,276	22,037
	Only immune	531	74	71	116	792
	Only Reproduction	6,131	47	2,536	1,946	10,660
	Without signal	8,310	179	3,119	2,194	13,802
	Total	27,161	575	10,023	9,532	47,291
	**2**^**nd **^**filtration**					
Reverse	Both systems	2,976	153	1627	5,493	10,249
	Only immune	298	85	86	257	726
	Only reproduction	2,332	61	1,360	3,112	6,865
	Without signal	9,059	359	4,812	15,221	29,451
	Total	14,665	658	7,885	24,083	47,291

It is remarkable that after the second filtration, 2,976 sequences (6.3%) still showed signal in both strands in both types of tissues (brain-hypophysis-gonad and immune tissues). These double hybridization signals could represent putative NATs found for the first time in the turbot transcriptome. miRNAs, are one of the most relevant short NATs classes and function as regulators of gene expression at the level of translation, with an essential input in developmental processes [111]. Due to their growing importance in regulating gene expression, several miRNA databases have been already created. In Table 11, we show a selection of ten miRNAs from those identified in the Turbot 3 database including their number of reads, which could be considered as a gross indicator of their expression level. To our knowledge, these miRNAs are the first to be identified in turbot. Further work is being carried out on the turbot database for developing a consistent bioinformatic pipeline for miRNA identification, as well as for their validation using a Q-PCR approach.

**Table 11:** Representative sample of miRNAs found in the Turbot 3 database. miRNAs were identified by Blasting Turbot 3 database sequences against the miRBase

**Contig number**	**Read number**	**Turbot 3 database annotation**	**miRBase accession name**	**miRBase annotation**
6,514	54	Early growth response 1	MI0020478	*Cricetulus griseus miR-29a stem-loop*
32,392	3	LSU rRNA	MI0022328	*Bos taurus miR-6529 stem-loop*
1,984r	97	Serine/threonine-protein phosphatase 2A	MI0014190	*Homo sapiens miR-3160-2 stem-loop*
34,898r	2	Similar to dynamin 3	MI0000247	*Mus musculus miR-204 stem-loop*
49,897	2	Similar to Elongation factor 1-gamma	MI0015615	*Ciona intestinalis miR-4064 stem-loop*
40,442	3	Similar to ORFa	MI0019438	*Oryzias latipes miR-133-2 stem-loop*
40,442r	3	Similarity to transposases	MI0001124	*Oryza sativa miR169i stem-loop*
10,002r	1	Spindle and kinetochore-associated protein 2-like	MI0018844	*Anolis carolinensis miR-301a stem-loop*
8,914r	22	Unknown	MI0019711	*Glycine max miR5769 stem-loop*
10,002	1	Unnamed protein product	MI006276	*Homo sapiens miR-1183 stem-loop*

## Conclusions

This is the first time that the transcriptome of the reproductive and the immune systems of turbot have been widely explored together. Both systems are essential for the survival of individuals and are of primary importance for commercial aquaculture. This study was designed to fill in the gap of genomic resources in turbot and therefore to improve available turbot sequence databases, specifically in genes related to reproduction. The large amount of generated sequences (52,427 putative transcripts) resulted in one of the most complete available databases for flatfish, with more than half of the resources annotated by both gene and functional category. The detailed and focused sequence assembly and gene annotation strategies allowed the identification of several genes involved in the immune and the reproductive systems, being most of them involved in key functions. A large amount of genetic markers was identified, providing new tools for genomic studies. The performance of an informative pilot microarray was assessed and identification of putative miRNAs was possible. Thus, NGS technologies represent an essential tool to increase exponentially genomic resources in non-model species, opening new insights for our understanding of key biological processes and addressing production bottlenecks in their aquaculture.

## Methods

Animals were treated according to the Directive 2010/63/UE of the European Parliament and of the Council of 22 September 2010 on the protection of animals used for experimentation and other scientific purposes. All experimental protocols were approved by the Institutional Animal Care and Use Committee of the University of Santiago de Compostela (Spain).

### Sanger sequencing

#### Experimental design and samplings

The *E. scophthalmi* infection trial was performed at the facilities of CETGA (Centro Tecnológico Gallego de Acuicultura; NW Spain). Naïve turbot (8484 recipients = R and 8484 controls = C) from a balanced mixture of five unrelated families with known pedigree, hatched and reared at a commercial fish farm were sent to CETGA facilities and acclimated to experimental conditions for 10 days before the beginning of the experiment. R and C fish were kept in separate tanks (7 tanks for each group) in two separated recirculating systems, with constant water temperature (19–20°C) and fed with commercial dried pellets. R fish were infected by oral intubation with intestinal scrapings containing *E. scophthalmi* stages obtained from infected turbot, for two consecutive days. C fish were maintained under equivalent conditions as R fish, but intubated with PBS instead. More details on this procedure can be found in a previous work [112].

The progression of the infection was monitored by sampling both C and R groups at different times post inoculation (p.i.). The prevalence of infection at each sampling point was obtained by detecting positive fish by either PCR or histology in any of the organs examined. At each sampling point, 14 fish from each group were sized, weighed and euthanized by over-exposure to benzocaine (3-aminobenzoic acid ethyl ester, 100 mg L-1, Sigma, St. Louis, MO, USA). The resulting prevalence of infection was 0, 7.1, 28.6, 85.7 and 92.9% at 4, 7, 14, 25 and 34 days p.i, respectively. No C fish was found to be infected.

Samples of spleen, head kidney, thymus, liver and pyloric caeca were rapidly dissected, immediately frozen in liquid nitrogen and stored at −80°C until used for RNA extraction. At each sampling time, samples of each tissue from the different individual fish from each group (C, R) were pooled. The serial times of sampling provided tissues expressing different genes related to immune response from initial until late states of the infection.

#### RNA isolation, library preparation and sequence analysis

RNA extraction of samples from control and infected fish, cDNA library construction and sequencing were performed as described elsewhere [36]. Briefly, RNA was extracted using TRIZOL Reagent (Invitrogen, Carlsbad, CA, USA). Poly-A mRNA was isolated using the Dynabeads® mRNA Purification Kit (Invitrogen, Carlsbad, CA, USA). The two cDNA libraries (control and infected) were directionally constructed (5^′^*EcoR*I, 3^′^*Xho*I), with equal amounts of RNA from each tissue at each sampling time, using the ZAP-cDNA Library Construction Kit (Stratagene, La Jolla, CA, USA), except size fractioning that was performed with the SizeSep 400 Spun Columns (GE Healthcare, Uppsala, Sweden). Plasmid DNA was isolated from approximately 4,000 clones from each library using the DirectPrep® 96 Miniprep kit (QIAGEN, Valencia, CA, USA). Plasmid DNA was sequenced following the ABI Prism BigDye™ Teminator v3.1 Cycle Sequencing Kit protocol on an ABI 3100 DNA sequencer (Applied Biosystems, Foster City, CA, USA). All clones were sequenced from their 3^′^ ends using a standard T7 primer to obtain the highest specific gene sequences for oligo-microarray design. Those clones that suffered a systematic drop on sequencing signal after poly-A tails were sequenced from the 5^′^ end. Basecalling from chromatogram traces was performed by using PHRED [113,114].

### 454 pyrosequencing run

#### Reproductive tissue sampling and RNA extraction

A total of 30 turbot samples were collected from CETGA from a mixture of unrelated genetic families. In order to obtain the widest possible range of expressed transcript sub-sets, tissues were dissected in fish at different stages of gonad development. The number, age and the mean values of biometry (standard length and body weight) for each animal group were the following: undifferentiated animals (n = 5; 90 days post hatch [dph], 5.2 ± 0.6 cm, 2.9 ± 0.9 g); differentiating animals (n = 4; 150 dph, 9.8 ± 1.3 cm, 21.8 ± 9.5 g); male juveniles (n = 4; 400 dph, 20.9 ± 4.2 cm, 195.0 ± 123.0 g); female juveniles (n = 5; 450 dph, 23.4 ± 3.6 cm, 264.1 ± 93.4 g); male broodstock (n = 3; 900 dph, 44.8 ± 3.6 cm, 2,059.0 ± 591.6 g) and female broodstock (n = 3; 900 dph, 47.7 ± 6.4 cm, 2,834.3 ± 1,264.9 g). Brain and hypophysis from broodstock animals were also dissected and rapidly flash frozen in liquid nitrogen. Gonads were fully isolated in adult and juvenile fish and thus gonadal tissue was devoid of any other tissue. However, gonads of sexually differentiating fish contained a bit of attached epithelium. Due to their extremely small size, the isolation of the gonads alone was not feasible and thus samples contained also portions of the surrounding tissues.

RNA was individually extracted by RNeasy Mini Kit (Qiagen, Hilden, Germany) following the manufacturer’s instructions. Quantity was determined using a Nanodrop spectrophotometer (Nanodrop Technologies, US). The RNA integrity number (RIN) was determined in an Agilent BioAnalizer (Agilent Technologies, US). RNA samples with a RIN > 8.1 were further processed for the sequencing run. A pooled sample was generated by mixing 70% of gonads containing equal amounts of RNA from each individual and 30% of equal amount of RNA from broodstock brains and hypophysis tissues.

#### cDNA library, normalization and 454 FLX Titanium pyrosequencing

Full-length-enriched double stranded cDNA was synthesized from 1.5 μg of pooled total RNA using the MINT cDNA synthesis kit (Evrogen, Moscow, Russia) according to the manufacturer’s protocol, and was subsequently purified using the QIAquick PCR Purification Kit (Qiagen USA, Valencia, CA). The amplified cDNA was normalized using the Trimmer kit (Evrogen, Moscow, Russia) to minimize differences in representation of transcripts [115,116]. The single-stranded cDNA fraction was then amplified twice by sequential PCR reactions according to manufacturer’s protocol. Normalized cDNA was purified using the QIAquick PCR Purification Kit (Qiagen USA, Valencia, CA). Normalized cDNA (5 μg) was used to generate a 454 library. cDNA was fractionated into small, 300 to 800 bp fragments and the specific A and B adaptors were ligated to both the 3^′^ and 5^′^ ends of the fragments and used for purification, amplification, and sequencing steps. Two and a quarter PTP regions were used for the GS-FLX sequencing run using Titanium chemistry. All reagents and protocols were from Roche 454 Life Sciences, USA. 454 data was processed with Roche’s software, using default settings, to obtain fasta and quality files containing the trimmed sequence of all reads [117]. Contigs with at least 100 bp were recovered. Sequences were *de novo* assembled into contigs by running Mira v3.2.0rc1 in *EST* mode. Contigs less than 100 bp were filtered out and the rest was blasted against *D. rerio* RefSeq protein sequences with est2assembly’s analyse_assembly.pl script [118] in order to validate the whole process.

### Turbot databases

Bioinformatic tools were developed to process all sequencing data obtained from both Sanger and 454 FLX Titanium technologies. The starting point of the current work was the Turbot 1 database, which was reported previously [36]. In order to generate the Turbot 2 database sequences of Turbot 1 database (9,873) were clustered with: 3,043 sequences obtained from the *E. scophthalmi* trial cDNA libraries, 1,371 genomic sequences from enriched DNA libraries [51] and 3,339 sequences available in public databases [52], using CAP3 software (http://seq.cs.iastate.edu/) (Table 1). The resulting “.ace” file was used to study coverage and construct user-friendly alignment views with Mview [119]. To construct the Turbot 3 database, the primitive sequences of Turbot 2 (17,626) were pooled with the 454 contigs (65,472) and then clustered using CAP3 software. The resulting contigs and singletons were annotated using AutoFact (http://www.bch.umontreal.ca/Software/AutoFACT.htm), BLASTN and BLASTX with databases nr, UniProt, UniRef, COG, KEGG, PFam, LSU and SSU. Results were uploaded to a MySQL database and a portal web was created.

To study the different pathways found in the Turbot 3 database the DAVID web tool was used [120,121]. After the selection of the pathways of interest, a list of reference genes was downloaded from the NCBI RefSeq database and BLASTed against the Turbot 3 database. A gene was considered present in our database if its reference sequence had a match with an e-value cut off ≤ 1,00E-5 and hit length ≥50. To make the colour pathway diagrams (Additional file 2 and Additional file 4) the KEGG mapper tool http://www.genome.jp/kegg/tool/map_pathway2.html was used [122,123]. Due to the lack of a *D. rerio* Chemokine signaling pathway in KEGG website the human version was used for Additional file 2. In Additional file 4, the Progesterone-mediated oocyte maturation pathway from *D. rerio* given by KEGG website is labeled as *Xenopus* oocyte. This label is kept in the figure.

### Microsatellites and SNPs

For SSR and SNP detection, EST sequences were clustered with CAP3 using default parameters and the resulting “.ace” format assembly file was fed into the corresponding programs. The set of unique sequences was searched for microsatellites using the SPUTNIK program (http://espressosoftware.com/sputnik/). The minimum repeat number used for this search was six for dinucleotide and four for tri-, tetra- and pentanucleotide microsatellites. Microsatellite-containing ESTs were identified as candidates for marker development if they presented enough flanking sequences on either side of the repeats for primer design. Whenever possible, we selected three putative primers using the Primer3 software (http://frodo.wi.mit.edu/primer3/).

SNP detection was performed with contigs of at least four sequences using the QualitySNP program (http://www.bioinformatics.nl/tools/snpweb/). This program uses three filters for the identification of reliable SNPs (see [92] for details). SNPs that pass filters 1 and 2 are called real SNPs and those passing all filters are called true SNPs. The resulting files were processed with our own custom Perl programs to extract relevant information. The obtained true SNPs were imported into a MySQL database (http://www.mysql.com). A user-friendly web access interface was designed so that contig graphs are clickable and the output visually refined with color-coded nucleotide views (http://bio-mview.sourceforge.net/). A graphical interface allowing for SNP database search by alleles, contig depth, and annotation was also established in our on line database. Searchable chromatograms for each of the Sanger sequences making up each contig were also included. It should be emphasized that SNPs detected with the help of bioinformatic pipelines are only putative and they should be technically validated.

To ensure identification of new molecular markers, sequences similar to GenBank deposited sequences were filtered out to avoid identification of already known SSR and SNP sequences, especially the ones previously identified by turbot [18,19,48,51,94,124,125].

### Pilot-microarray platform

A custom 2 x 105 K array was printed with turbot sequences from the Turbot 3 database by Agilent Technologies (US). In order to study the orientation of the non-annotated sequences and their possible gene expression, false annotation of genes and identify possible NATs, oligos were designed in both orientations, forward and reverse. Oligo design was done by using Repeat Masker to eliminate low-complexity regions, and then OligoArray 2.1 software to do the design itself [126]. Cross-hybridization between oligos was checked by BLAST searches against the entire Turbot 3 database and oligos with ≥ 3 putative cross-hybridizations were removed. A total number of 96,292 oligos were printed and almost half of the array contained oligos (47,291) also designed with the opposite orientation. This pilot microarray also included all default positive and negative controls defined by the company (1,325 spots).

### Microarray hybridization

The same samples of immune tissues used for library construction and Sanger sequencing and those from the brain-pituitary-gonad axis used for 454 sequencing were used for hybridization (in duplicate) with the pilot microarray. A total of four microarrays were used, two for the reproductive system and two for the immune system. Hybridizations were performed at the Universidad de Santiago de Compostela (USC) Functional Genomics Platform by the Agilent Technology Gene Expression Unit using a 1-colour labeling protocol. This method demonstrated very similar performances to the 2-colour protocol [97,127]. Briefly, 50 ng of total RNA were labelled using the Low Input Quick Amp Labeling Kit, One-Color (Cy3) (Agilent Technologies, USA). cRNA was prepared for overnight hybridization with the corresponding buffers during 17 h at 65°C and washed on the following day. Hybridized slides were scanned using an Agilent G2565B microarray scanner (Agilent Technologies, USA).

### Pilot microarray data processing, filtration, and identification of NATs

The hybridization signal was captured and processed using an Agilent scanner (G2565B, Agilent Technologies, USA). The scanner images were segmented with the Agilent Feature Extraction Software (v9.5) using protocol GE1-v5_95. Extended dynamic range implemented in the Agilent software was applied to avoid saturation in the highest intensity range. Agilent feature extraction produced the raw data for further pre-processing. The processed signal (gProcessed-Signal) value was chosen as statistical for the absolute hybridization signal.

The filtration process was made in two steps. First, the features which did not conform with any of the following well established quality criteria were filtered: (1) non-uniform pixel distributed outliers and population replicate outliers according to the default Agilent feature extraction criteria; (2) features whose ratio between processed signal and their error was below 2; (3) spots not differentiated from background signal (as estimated for each spot); (4) features below the limit where the linear relationship between concentration and intensity was lost according to Spike-In information. The numbers of oligos filtered using this first step is shown in Table 10. Second, two additional filtering criteria were applied: (5) only features with intensity ≥ 100 fluorescence units were kept; (6) features likely to present cross-hybridization were filtered. Table 10 shows the numbers of oligos filtered using the complete filtration process.

For miRNA identification in the Turbot 3 database, a BLASTN search against the miRBase v.18 database (http://www.mirbase.org/) was used. The ten best matches were selected and are depicted in Table 11.

Statistical analyses were carried out with the statistical language R (2.13.1 version). The GOStats Bioconductor package (version 2.18) was used to perform the analysis of GO Terms.

## Competing interests

The authors declare that they have no competing interests.

## Authors’ contributions

FP and PM lead and supervised the study. *E. scophthalmi* experimental design, parasite diagnosis and collection of immune-related tissues were done by MQ and ASB, and cDNA library constructions and Sanger sequencing data by BGP. Gonad sampling design and RNA extraction was performed by FP and LR, respectively. 454 sequencing run data was obtained from the Centres Científic i Tecnològics de la Universitat de Barcelona (CCiT-UB) by JVP. JAD compiled the Turbot 2 and Turbot 3 databases. CF designed the oligo microarray and AGT processed microarray results. Microarray hybridizations were performed by BGP. The paper was mainly written by LR, also contributed by BGP, JAD, AGT, JVP, ASB, and FP, and revised and edited by FP and PM. All authors read and approved the final manuscript.

## Supplementary Material

Additional file 1 List of all the immune-related genes found in the Turbot 3 database. Click here for file

Additional file 2 **Chemokine signaling pathway representing the present (in blue) and absent genes (without color) in the Turbot 3 database.***ADCY 1*, *2*, *3*, *4*, *5*, *7*, *9*: adenylate cyclase; *ADRBK 1*, *2*: adrenergic, beta receptor kinase; *AKT 1*, *2*, *3*: v-akt murine thymoma viral oncogene homolog; *RHOA*: ras homolog family member A; *ARRB 1*, *2*: arrestin beta; *BRAF*: v-raf murine sarcoma viral oncogene homolog; *CDC42*: cell division cycle 42 (GTP binding protein); *CCR4*: chemokine (C-C motif) receptor 4; *CRK*: v-crk sarcoma virus CT10 oncogene homolog; *DOCK2*: dedicator of cytokinesis 2; *PTK2B*: PTK2B protein tyrosine kinase 2 beta; *FGR*: Gardner-Rasheed feline sarcoma viral (v-fgr) oncogene homolog; *FOXO3*: forkhead box O3; *GNAI 1*, *2*, *3*: guanine nucleotide binding protein (G protein) alpha inhibiting activity polypeptide; *GN B1*, *B2*, *B3*, *G3*, *G10*: guanine nucleotide binding protein (G protein) beta polypeptide; *CXCR 1*, *2*, *3*, *4*: chemokine (C-X-C motif) receptor 3; *GRK 4*, *5*, *6*: G protein-coupled receptor kinase; *GRB2*: growth factor receptor-bound protein 2; *GSK 3A*, *3B*: glycogen synthase kinase 3 alpha; *HCK*: hemopoietic cell kinase; *HRAS*: v-Ha-ras Harvey rat sarcoma viral oncogene homolog; *IKBK B*, *G*: inhibitor of kappa light polypeptide gene enhancer in B-cells kinase; *IL8*: interleukin 8; *JAK2*: Janus kinase 2; *KRAS*: v-Ki-ras2 Kirsten rat sarcoma viral oncogene homolog; *LYN*: v-yes-1 Yamaguchi sarcoma viral related oncogene homolog; *NRAS*: neuroblastoma RAS viral (v-ras) oncogene homolog; *PAK1*: p21 protein (Cdc42/Rac)-activated kinase 1; *PF4*: platelet factor 4; *PIK3CA3R 1*, *2*, *3*, *5*: phosphatidylinositol-4,5-bisphosphate 3-kinase, catalytic subunit alpha; PLCB 1, 2, 3, 4: phospholipase C beta; *PRKAC A*, *B*, *G*: protein kinase, cAMP-dependent catalytic; *PRKC B*, *D*, *Z*: protein kinase C; *MAPK 1*, *K3*: mitogen-activated protein kinase; *PRKX*: protein kinase, X-linked; *PXN*: paxillin; *RAC 1*, *2*: ras-related C3 botulinum toxin substrate; *RAF1*: v-raf-1 murine leukemia viral oncogene homolog 1; *RAP 1A 1B*: member of RAS oncogene family; *RELA*: v-rel reticuloendotheliosis viral oncogene homolog A; *SOS1*: son of seven less homolog 1; *SRC*: v-src sarcoma (Schmidt-Ruppin A-2) viral oncogene homolog; *STAT 1*, *3*, *5B*: signal transducer and activator of transcription, 91 kDa; *VAV1*: vav 1 guanine nucleotide exchange factor; *WAS L*: Wiskott-Aldrich syndrome (like); *ROCK2*: Rho-associated coiled-coil containing protein kinase 2; *BCAR1*: breast cancer anti-estrogen resistance 1; *ELMO1*: engulfment and cell motility 1; *RASGRP2*: RAS guanyl releasing protein 2 (calcium and DAG-regulated); *GNB 4*, *5*: guanine nucleotide binding protein (G protein) beta; *SHC2*: SHC (Src homology 2 domain containing) transforming protein 2; *GNG13*: guanine nucleotide binding protein (G protein) gamma 13; *PARD3*: par-3 partitioning defective 3 homolog; *PREX1*:phosphatidylinositol-3,4,5-trisphosphate-dependent Rac exchange factor 1. Click here for file

Additional file 3 List of all the reproduction-related genes found in the Turbot 3 database. Click here for file

Additional file 4 **Progesterone-mediated oocyte maturation pathway representing the present (in blue) and absent genes (without color) in the Turbot 3 database.***CDC 16*, *23*, *25*, *26*, *27*: cell division cycle; *HSP90 AA1.1*, *AA1.2*, *AB1*: heat shock protein 90 alpha (cytosolic); *MAPK 1B*, *3*, *8A*, *8B*, *9*, *10*, *11*, *12A*, *12B*, *13*, *14A*, *14B*: mitogen-activated protein kinase; *ZORBA*: Orb/CPEB-related RNA-binding protein; *RAF 1A*, *1B*: v-raf-1 murine leukemia viral oncogene homolog; *CCN A1*, *B1*, *B2*, *B3*: cyclin; CDK 1, 2: cyclin-dependent kinase; *IGF1R A*, *B*: insulin-like growth factor 1 receptor; *PLK1*: polo-like kinase 1; *ANAPC 2*, *5*, *11*: anaphase promoting complex subunit; *GNA I1*, *IA*, *I2*, *2 L*: guanine nucleotide binding protein (G protein) alpha inhibiting activity; *RPS6K A1*,*A2*, *A3A*, *AL*: ribosomal protein S6 kinase like; *AKT 2*, *2 L*, *3A*: v-akt murine thymoma viral oncogene homolog; *FZR1*: fizzy/cell division cycle 20 related 1; *PRKAC AA*, *BA*, *AB*, *BB*, *CB*: protein kinase cAMP-dependent catalytic; *ARAF*: v-raf murine sarcoma 3611 viral oncogene homolog; *BRAF*: v-raf murine sarcoma viral oncogene homolog; *PIK3R 2*, *3B*: phosphoinositide-3-kinase regulatory subunit; *MAP2K1*: mitogen-activated protein 2 kinase 1; *KRAS*: v-Ki-ras2 Kirsten rat sarcoma viral oncogene homolog; *MAD2 L1 2B-like*: *MAD2* mitotic arrest deficient; *ADCY 1A*, *1B*, *3C*, *6B*, *7*: adenylate cyclase; *PGR*: progesterone receptor; *GNAI3*: guanine nucleotide binding protein (G protein) alpha inhibiting activity polypeptide 3. Click here for file
