# Supplementary figures and images for: A combined strategy involving Sanger and 454 pyrosequencing increases genomic resources to aid in the management of reproduction, disease control and genetic selection in the turbot (Scophthalmus maximus)
Source: BMC Genomics. 2013 Mar 15;14:180. doi: 10.1186/1471-2164-14-180 (PMC3700835; doi:10.1186/1471-2164-14-180)

## Slide 1
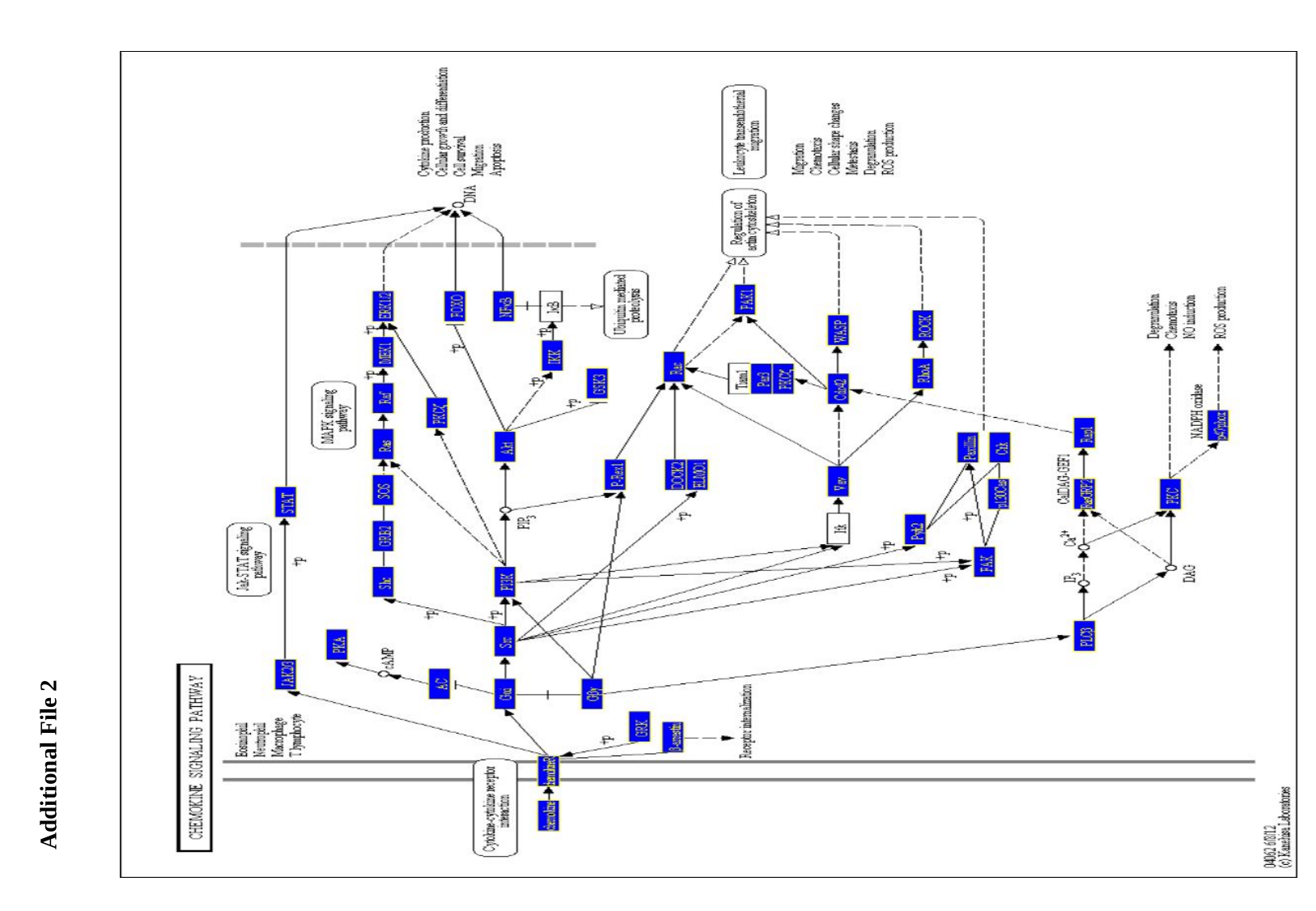

Additional File 2

Supplement: Additional file 2 — Chemokine signaling pathway representing the present (in blue) and absent genes (without color) in the Turbot 3 database.ADCY 1, 2, 3, 4, 5, 7, 9: adenylate cyclase; ADRBK 1, 2: adrenergic, beta receptor kinase; AKT 1, 2, 3: v-akt murine thymoma viral oncogene homolog; RHOA: ras homolog family member A; ARRB 1, 2: arrestin beta; BRAF: v-raf murine sarcoma viral oncogene homolog; CDC42: cell division cycle 42 (GTP binding protein); CCR4: chemokine (C-C motif) receptor 4; CRK: v-crk sarcoma virus CT10 oncogene homolog; DOCK2: dedicator of cytokinesis 2; PTK2B: PTK2B protein tyrosine kinase 2 beta; FGR: Gardner-Rasheed feline sarcoma viral (v-fgr) oncogene homolog; FOXO3: forkhead box O3; GNAI 1, 2, 3: guanine nucleotide binding protein (G protein) alpha inhibiting activity polypeptide; GN B1, B2, B3, G3, G10: guanine nucleotide binding protein (G protein) beta polypeptide; CXCR 1, 2, 3, 4: chemokine (C-X-C motif) receptor 3; GRK 4, 5, 6: G protein-coupled receptor kinase; GRB2: growth factor receptor-bound protein 2; GSK 3A, 3B: glycogen synthase kinase 3 alpha; HCK: hemopoietic cell kinase; HRAS: v-Ha-ras Harvey rat sarcoma viral oncogene homolog; IKBK B, G: inhibitor of kappa light polypeptide gene enhancer in B-cells kinase; IL8: interleukin 8; JAK2: Janus kinase 2; KRAS: v-Ki-ras2 Kirsten rat sarcoma viral oncogene homolog; LYN: v-yes-1 Yamaguchi sarcoma viral related oncogene homolog; NRAS: neuroblastoma RAS viral (v-ras) oncogene homolog; PAK1: p21 protein (Cdc42/Rac)-activated kinase 1; PF4: platelet factor 4; PIK3CA3R 1, 2, 3, 5: phosphatidylinositol-4,5-bisphosphate 3-kinase, catalytic subunit alpha; PLCB 1, 2, 3, 4: phospholipase C beta; PRKAC A, B, G: protein kinase, cAMP-dependent catalytic; PRKC B, D, Z: protein kinase C; MAPK 1, K3: mitogen-activated protein kinase; PRKX: protein kinase, X-linked; PXN: paxillin; RAC 1, 2: ras-related C3 botulinum toxin substrate; RAF1: v-raf-1 murine leukemia viral oncogene homolog 1; RAP 1A 1B: member of RAS oncogene fa [file 1471-2164-14-180-S2.pptx]

## Slide 1
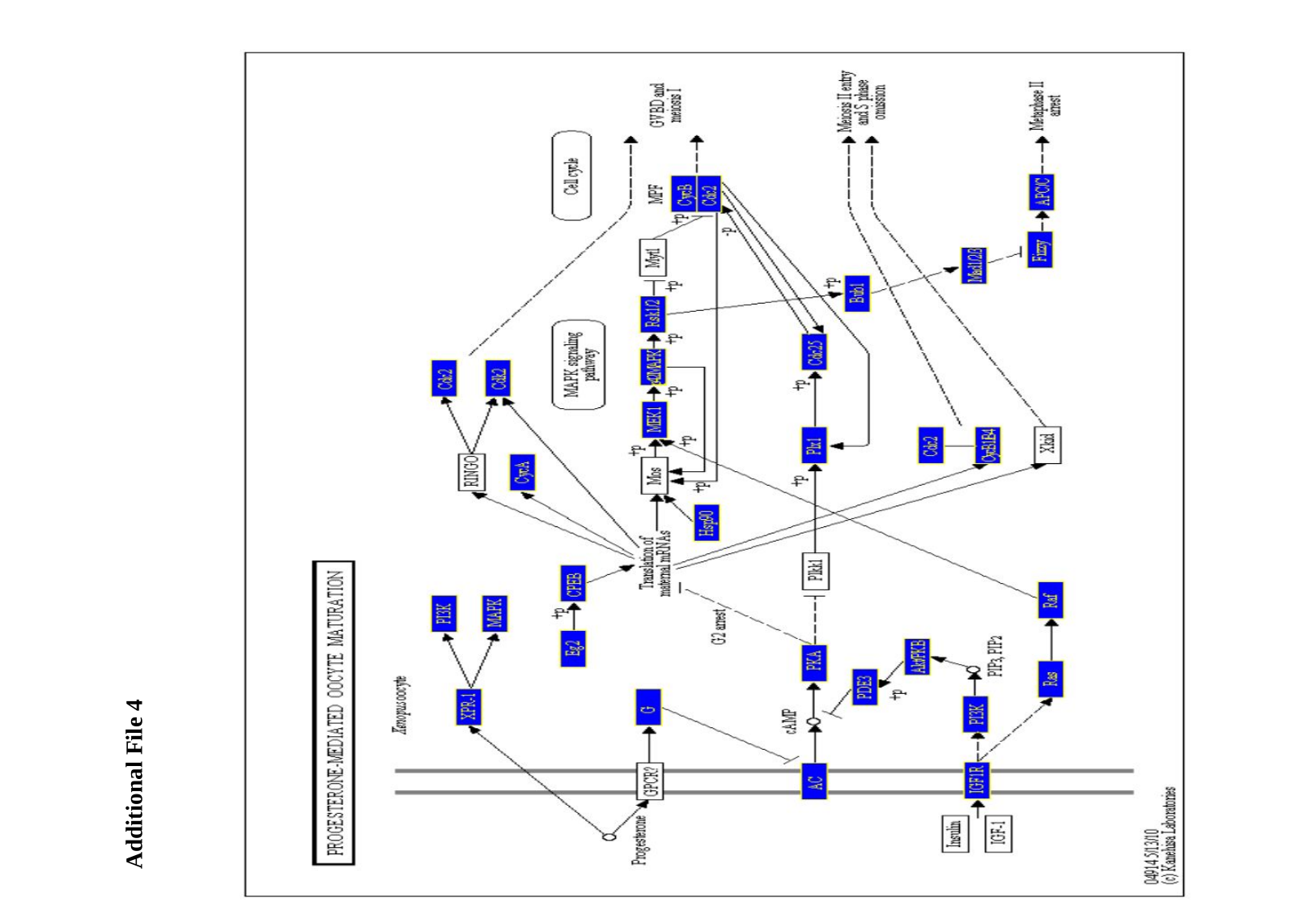

Additional File 4

Supplement: Additional file 4 — Progesterone-mediated oocyte maturation pathway representing the present (in blue) and absent genes (without color) in the Turbot 3 database.CDC 16, 23, 25, 26, 27: cell division cycle; HSP90 AA1.1, AA1.2, AB1: heat shock protein 90 alpha (cytosolic); MAPK 1B, 3, 8A, 8B, 9, 10, 11, 12A, 12B, 13, 14A, 14B: mitogen-activated protein kinase; ZORBA: Orb/CPEB-related RNA-binding protein; RAF 1A, 1B: v-raf-1 murine leukemia viral oncogene homolog; CCN A1, B1, B2, B3: cyclin; CDK 1, 2: cyclin-dependent kinase; IGF1R A, B: insulin-like growth factor 1 receptor; PLK1: polo-like kinase 1; ANAPC 2, 5, 11: anaphase promoting complex subunit; GNA I1, IA, I2, 2 L: guanine nucleotide binding protein (G protein) alpha inhibiting activity; RPS6K A1,A2, A3A, AL: ribosomal protein S6 kinase like; AKT 2, 2 L, 3A: v-akt murine thymoma viral oncogene homolog; FZR1: fizzy/cell division cycle 20 related 1; PRKAC AA, BA, AB, BB, CB: protein kinase cAMP-dependent catalytic; ARAF: v-raf murine sarcoma 3611 viral oncogene homolog; BRAF: v-raf murine sarcoma viral oncogene homolog; PIK3R 2, 3B: phosphoinositide-3-kinase regulatory subunit; MAP2K1: mitogen-activated protein 2 kinase 1; KRAS: v-Ki-ras2 Kirsten rat sarcoma viral oncogene homolog; MAD2 L1 2B-like: MAD2 mitotic arrest deficient; ADCY 1A, 1B, 3C, 6B, 7: adenylate cyclase; PGR: progesterone receptor; GNAI3: guanine nucleotide binding protein (G protein) alpha inhibiting activity polypeptide 3. [file 1471-2164-14-180-S4.pptx]
